# The LEAP (Learn to Eat at Peace) Eating Disorders Program: overview & evaluation

**DOI:** 10.1186/2050-2974-2-S1-O12

**Published:** 2014-11-24

**Authors:** Siew Soon, Peter O'Keefe

**Affiliations:** 1 The Geelong Clinic Eating Disorders Program, Melbourne, Australia; 2 Barwon Health Eating Disorder Service, Melbourne, Australia

## 

The Geelong Clinic's 40-day LEAP (Learn to Eat at Peace) Eating Disorders Inpatient Program is unique as consumers have had considerable input in the development and ongoing review of the program. The LEAP philosophy emphasises trust, dignity, individualised treatment, and skill acquisition. The program involves staff-supported meals and groups facilitated by a multidisciplinary team. The aim of this study was to evaluate if there were changes in patients' clinical symptoms following the program.

Participants were eating disorder (ED) patients (n = 43), aged 16 to 55 years. Data was collected through self-report measures at admission and discharge from the program. Repeated-measures ANOVAs were used to assess changes in variables including restraint over eating, eating concern, shape concern, weight concern, body mass index, depression, anxiety, and stress.

Results indicated statistically significant improvements across all variables. Effect sizes were large (partial eta-squared = .14 to .58), indicating clinical significance. At discharge, 85% of patients agreed that they were satisfied with therapy and with therapists.

Findings provide empirical evidence that following the LEAP Program, patients report significant improvements in ED symptoms and general psychopathology, and were satisfied with treatment. Findings support the importance of consumer involvement, using a collaborative approach, and multidisciplinary treatment of EDs.

This abstract was presented in the **Treatment in Community and Inpatient Settings** stream of the 2014 ANZAED Conference.

